# High-Fat Breakfast Meal Replacement in Overweight and Obesity: Implications on Body Composition, Metabolic Markers, and Satiety

**DOI:** 10.3390/nu11040865

**Published:** 2019-04-17

**Authors:** Abbie E. Smith-Ryan, Katie R. Hirsch, Malia N. M. Blue, Meredith G. Mock, Eric T. Trexler

**Affiliations:** 1 Applied Physiology Laboratory, Department of Exercise and Sport Science, The University of North Carolina, Chapel Hill, NC 27599, USA; ktrose23@live.unc.edu (K.R.H.); mnm3303@live.unc.edu (M.N.M.B.); mock.mere@gmail.com (M.G.M.); trexlere@live.unc.edu (E.T.T.); 2 Human Movement Science Curriculum, Department of Allied Health Science, University of North Carolina, Chapel Hill, NC 27599, USA

**Keywords:** overweight, obese, fat mass, metabolic rate, supplement, medium chain triglyceride

## Abstract

The purpose of this paper was to determine the effect of replacing breakfast with a high-fat drink on fat mass (FM), lean mass (LM), percent body fat (%BF), visceral fat (VAT), resting metabolic rate (RMR), fuel utilization (RER), blood lipids and satiety in overweight and obese adults. Healthy adults (*n* = 42; 21 Females; body mass index (BMI): 32.8 ± 4.6 kg·m^−2^) were randomized to control (CON; *n* = 21) or meal replacement (MRP; *n* = 22) groups. Body composition was measured using a four-compartment model; RMR and RER were assessed from indirect calorimetry. The MRP (70% fat) was consumed once daily for eight weeks. For males, there was no change (*p* > 0.05) in FM (mean difference (MD) = 0.41 ± 1.19 kg], %BF MD = 0.50 ± 1.09%, LM MD = −0.64 ± 1.79 kg, or VAT MD = −0.31 ± 1.36 cm for MRP versus CON. Similarly, no differences for females for FM MD = −0.73 ± 1.37 kg, %BF MD = −0.57 ± 1.26%, LM MD = 0.31 ± 1.37 kg, or VAT MD: −0.83 ± 1.2 cm. HDL was significantly reduced in the MRP group for females (adjusted mean change: −6.41 ± 4.44 units, *p* = 0.018). There was no effect on RMR or RER. Satiety increased in the afternoon for MRP (*p* = 0.021). Despite high fat, no negative impact on lipids resulted; increased satiety may be beneficial for controlling afternoon cravings, but does not affect body composition.

## 1. Introduction

While a consensus on whether skipping breakfast impedes [1] or enhances [2] weight loss has not been met, a growing body of literature is in agreement regarding the need for high quality breakfast choices to improve health outcomes [3]. High-fat diets are traditionally associated with the negative connotation of promoting fat gain. Recent literature has demonstrated that diets higher in fat can promote significant weight loss and improvements in body composition [4,5]. Specifically at breakfast the majority of available data has demonstrated a favorable effect of a higher- compared to lower-fat breakfast intake on postprandial glucose response [6] and reduced risk of type II diabetes [7,8]. The type of fat may also be important; rapid oxidation of medium-chain triglycerides (MCT) compared to long-chain triglycerides, has been shown to result in greater energy expenditure and fat oxidation in humans [9,10,11]. Replacement of long chain triglycerides with MCTs has been associated with modest changes in body weight and composition, with no adverse effects on lipids [5].

Consistent use of nutrient dense meal replacements have repeatedly shown to be an effective weight loss tool, second only to bariatric surgery for effectiveness [12]. Due to the high compliance and minimal behavior modification, use of a meal replacement may be a key strategy to induce changes in body composition and metabolic health [13,14]. Previous meta-analytical data has demonstrated liquid meal replacements to induce greater weight loss compared to traditional reduced calorie diets [15]. Liquid meal replacements at breakfast have also been supported for use in clinical populations to effectively control blood glucose and lipid levels [16].

To date, few studies have examined the effects of a high-fat liquid meal replacement at breakfast, as a minimal nutrition intervention on body composition and cardiometabolic health. With an increasing popularity for higher-fat, ketogenic style breakfasts, the current study took a pragmatic approach to evaluate the effects of consuming a high fat breakfast meal replacement (70% fat) for eight weeks on body composition, resting metabolic rate (RMR), and resting fuel utilization (RER) in overweight and obese men and women. Effects on blood lipids and feelings of satiety were also evaluated.

## 2. Materials and Methods 

### 2.1. Subjects

Approximately 114 individuals expressed interest in participation, 64 individuals qualified for in-person inclusion-exclusion review, and 56 individuals were initially enrolled in the study. Seven individuals withdrew from participation prior to testing, resulting in a total of 49 overweight and obese men (*n* = 23) and premenopausal women (*n* = 26) who participated in the study (full recruitment details are presented elsewhere [17]). An additional six participants withdrew from the study following baseline testing due to reasons including lack of time, lost to follow-up, gastrointestinal distress (*n* = 2 from the MRP). One subject was removed due to low supplement compliance (<50%). Therefore, 42 subjects (*n* = 21 men; *n* = 21 women) were analyzed for the present study. All subjects were between the ages of 18 and 55 years (Table 1) and considered healthy, having no history of medical or surgical events including cardiovascular disease, diabetes, renal, hepatic, or musculoskeletal disorders. Subjects were weight-stable (±4.5 kg) and had maintained consistent dietary habits (i.e., no changes in macronutrient or caloric intake) within three months prior to enrollment. This was determined from the self-report through a lifestyle questionnaire, as well as a one-on-one personal interview. Subjects were excluded from participation if they reported prior use of a meal replacement, dietary supplement, or inconsistent use of medication that may have influenced metabolism within eight weeks prior to enrollment. All subjects were instructed to maintain their normal physical activity and dietary habits throughout the study. This trial was registered on clinicaltrials.gov prior to the study initiation: NCT02482545.

### 2.2. Experimental Design

In a randomized, delayed-controlled intervention, individuals were randomized to control (CON; *n* = 21) or meal replacement (MRP; *n* = 21) treatment groups. Prior to testing, subjects provided written informed consent approved by the University’s Institutional Biomedical Review Board, completed a health history questionnaire, and received basic dietary recommendations based on a four-day dietary log (two weekdays, two weekend days). A two-week run-in period preceded the first testing session to allow any potential changes in dietary changes that may occur at the beginning of a study to normalize [18]. Resting measures of metabolic rate and substrate utilization, body composition, and blood samples were collected in the morning, eight hours fasted. A steady-state cardiorespiratory test was performed an average of 24 h following resting measures (0–72 h), at least four h fasted. All subjects completed a satiety questionnaire (Satiety Labeled Intensity Magnitude (SLIM)). Over the eight-week intervention, the MRP group consumed one scoop of MRP every morning while the CON group maintained their normal dietary habits. All subjects completed three-day dietary food logs biweekly throughout the intervention period and measures were repeated after eight weeks.

### 2.3. Body Composition

A gold-standard, four-compartment (4C) model was used to determine fat mass (FM (kg) = 2.748(BV) − 0.699(TBW) + 1.129(Mo) − 2.051(BM)), percent body fat (%BF = (FM/BM) × 100), and lean mass (LM (kg) = BM − FM) [19,20]. Where fat mass = FM; body volume = BV; total body water = TBW, total body bone mineral density = Mo, body mass = BM, percent fat = %BF, and lean mass = LM. Body volume was measured using the air-displacement plethysmographys (BodPod^®^, COSMED USA, Inc., Concord, CA, USA), following manufacturer recommendations, and determined from the average of two measurements; lung volume was predicted from the default software (Software Version 5.4.1, COSMED USA, Inc., Concord, CA, USA). Total body water (TBW) was measured as the average of two measurements using the bioelectrical impedance spectroscopy (BIS; SFB7, ImpediMed, Queensland, Australia) following manufacturer recommendations for lead and electrode placement on the wrist and ankle. The bone mineral content (BMC) was measured using dual-energy X-ray absorptiometry (DEXA; GE Lunar iDXA, GE Medical Systems Ultrasound & Primary Care Diagnostics, Madison, WI, USA) and used to calculate the total body bone mineral density (Mo = BMC (kg) × 1.0436). Total body DEXA scans were performed by a trained DEXA technician, following manufacturer recommendations, and analyzed using the default software (enCORE Software Version 16). Six individuals were wider than the scanning parameter and left limbs were estimated from the right limbs in accordance with manufacturer recommendations. For all body composition tests, subjects wore lightweight athletic clothing and removed all metal items that may disrupt the measures. Test-retest reliability for the 4C model from our lab in a similar population are as follows: FM intraclass correlation coefficient (ICC) = 0.994, standard error of measure (SEM) = 0.830 kg, and minimum difference (MD) = 2.30 kg, %BF ICC = 0.988, SEM = 0.868%, MD = 2.40%, and LM ICC = 0.996, SEM = 0.842 kg, MD = 2.33 kg.

**Figure 1:**
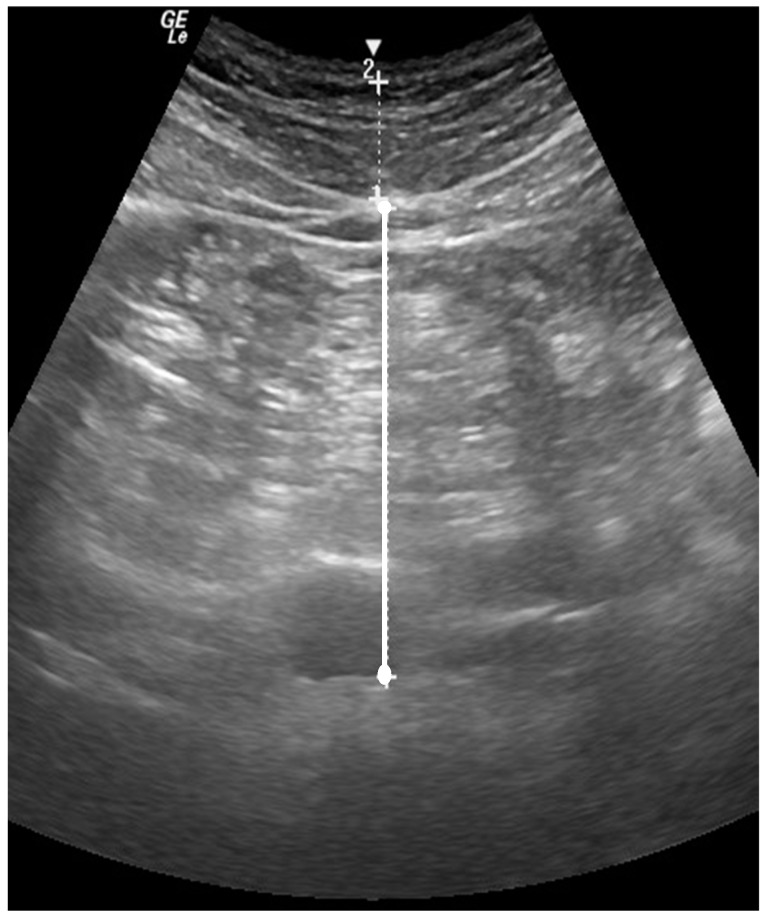
B-mode US image of the abdomen, 5 cm proximal to the umbilicus. The perpendicular distance between the interior border of the rectus abdominis and the posterior wall of the aorta was quantified as VAT.

Visceral fat (VAT) was quantified using the brightness-mode (B-mode) ultrasound (GE LOGIQ-e, Software version R8.0.7, GE Healthcare, Madison, WI, USA) with standardized settings (Frequency: 4.0 MHz, Gain: 45). The wide-band convex array ultrasound transducer (GE: C1-5 RS) with transducer gel was positioned at approximately 5 cm proximal to the umbilicus and a still image of the abdomen was captured at the moment of complete exhale (Figure 1). Visceral fat was quantified as the perpendicular distance between the interior border of the rectus abdominus and the posterior wall of the aorta [21,22]; the average of two measures was recorded as VAT. Test-retest reliability for VAT produced an ICC = 0.99, SEM = 0.35 cm, and MD = 0.69 cm.

### 2.4. Resting Metabolic Test

Estimations of RMR and resting RER were evaluated using a ventilated canopy with indirect calorimetry. Respiratory gases were collected and analyzed breath-by-breath for 30 min with a metabolic cart (TrueOne 2400, ParvoMedics, Inc., Sandy, UT, USA). The first 5 min of the test was excluded to allow breathing rate to normalize and allow for the adjustment of the dilution rate; RMR and RER were averaged over the remaining 25 min. The test-retest reliability produced a RMR ICC = 0.94, SEM = 125.6 kcal·day^−2^, and MD = 244.3 kcal·day^−2^, and a RER ICC = 0.83, SEM = 0.03 arbitrary units (a.u.), and MD = 0.05 a.u.

### 2.5. Steady-State Cardiorespiratory Test

Submaximal RER (RER_ex_) was evaluated during a 20 min steady-state cardiorespiratory test on a cycle ergometer (Lode, Gronigen, The Netherlands) [23,24]. Following a three-minute warm-up at a workload of 20 watts and a self-selected speed, the workload was increased until the heart rate (HR) reached 50−60% of heart rate reserve (HRR) as determined by the Karvonen formula [25]. Workload was adjusted throughout the test, as necessary, to maintain an appropriate HRR. Respiratory gases were analyzed via the indirect calorimetry (TrueMax 2400^®^, Parvo Medics, Salt Lake City, UT, USA), and RER_ex_ was recorded as the average of the 20 min test. To assess the metabolic flexibility, the change in RER (ΔRER) was calculated as the difference between RER_ex_ and resting RER.

### 2.6. Fasted Blood Sample 

A 4-ml sample from the antecubital region of the arm was collected and analyzed for the fasting total cholesterol (TC), high-density lipoproteins (HDL), low-density lipoproteins (LDL), triglycerides (TRG), and glucose (GLUC). Samples were analyzed by the UNC Biobehavioral Lab (Chapel Hill, NC, USA) using commercially available enzymatic assays. Blood samples were analyzed for lipid profiles within 15 min of the draw. The coefficient of variation was below 15% for all assays.

### 2.7. Questionnaires

Changes in satiety were evaluated using the Satiety Labeled Intensity Magnitude (SLIM) questionnaire in which subjects were asked to rank their degree of hunger/fullness right after breakfast, a few hours after breakfast, and during the afternoon [26].

### 2.8. Dietary Analysis

The nutrition analysis software (The Food Processor, version 10.12.0, Esha Research, Salem, OR, USA) was used to analyze all dietary food logs for the average calorie (CAL), carbohydrate (CHO), fat (FAT), and protein (PRO) intake; CHO/(PRO + FAT) ratio was calculated separately. Subjects completed dietary logs bi-weekly, completing four, three-day dietary logs over the course of the eight-week intervention period.

### 2.9. Supplementation

Subjects were randomly assigned to either the MRP or CON group using equal block randomization from Random Allocation Software (Isfahan, Iran). The MRP group was given a 5 lb tub of MRP and instructed to consume one scoop of MRP powder mixed with water every morning within an hour of waking, in replacement of normal breakfast. The MRP was 27g (155kcal), 6g carbohydrate (CHO;15%) − (3g CHO; 3g Fibersol^®^), 12.5g FAT (70%), 6.5g protein (PRO; 16%). As a note, the scientifically determined energy value for Fibersol is 1.6 kcal/gram. Ingredients included: Medium-chain triglyceride oil creamer, whey protein concentrate, cocoa powder, coconut oil creamer, milk protein isolate, maltodextrin, natural flavor, xanthan gum, salt, swerve^®^, lactase. The CON group was instructed to maintain normal dietary habits. Subjects recorded on dietary logs days in which the MRP was consumed throughout the intervention and tubs of MRP were returned and weighed at the end of the intervention to monitor compliance. On average, individuals consumed more than 90% of the MRP they were expected to consume, with consumption reported on an average of 53 of the expected 56 days. 

### 2.10. Statistical Analysis

A modified intention to treat population was evaluated. Subjects only present for the baseline visit were not included in the analyses since they did not have any data following consumption of the product. Differences between groups (MRP vs. CON) at post-testing for body composition, metabolism, and blood markers were analyzed using separate analysis of covariance (ANCOVA) with baseline scores used as the covariate for males and females, respectively. The homogeneity of regression assumption for ANCOVA was met for all variables using the custom homogeneity variance model combined with the Levene’s tests of equality of error variances. All post-hoc comparisons were Bonferroni adjusted. Dietary data was analyzed using separate two-way mixed factorial ANOVAs (group (MRP vs. CON) × time (base vs. wk 2 vs. wk 4 vs. wk 6 vs. wk 8)) to identify any group × time interactions. If a significant interaction was observed, the statistical model was decomposed by examining the simple main effects with one-way repeated measures ANOVAs for each group and one-way factorial ANOVAs for each time. In the event of simple main effects, Tukey post-hoc comparisons were performed among the groups, all pair-wise comparison dependent samples t-tests with Bonferroni corrections were performed across time. If there was no interaction, main effects were analyzed by collapsing across the non-interactive variable as described above for simple main effects. The level of significance was set at *p* ≤ 0.05. Descriptive statistics are presented as mean ± standard deviation and effect sizes as (ηp^2^). Raw values and adjusted change scores (week eight minus baseline) for all dependent variables are displayed. Analyses were performed using SPSS (Version 21, IBM, Armonk, NY, USA). 

## 3. Results

Forty-three individuals completed the study; one participant had low compliance (<50%) and therefore was not included in the modified intention-to-treat analysis. Data from twenty-one males (*n* = 11 MRP; *n* = 10 CON) and females (*n* = 10 MRP; *n* = 11 CON), respectively, were reported. 

### 3.1. Body Composition

**Figure 2:**
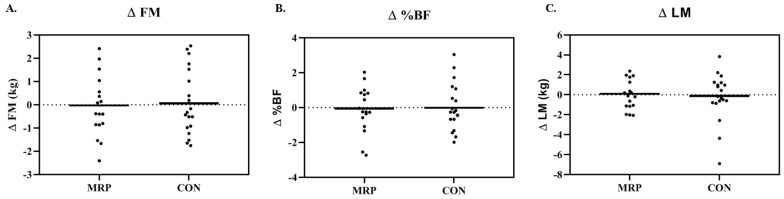
Individual responses for change in (**A**) fat mass (∆FM); (**B**) percent fat (∆%BF); and (**C**) lean mass (∆LM) for the entire sample in the meal replacement (MRP) and control (CON) groups.

Individual responses for change in FM, %BF, and LM for the entire sample are shown in Figure 2. For males, there was no significant difference in weight for the MRP group or CON group (mean difference (MD): −0.29 ± 1.37 kg; *p* = 0.682) at post-testing. Neither FM nor %BF resulted in a difference for MRP or CON (MD: FM: 0.41 ± 1.19 kg; *p* = 0.503, ηp^2^ = 0.038; MD: %BF: 0.50 ± 1.09%, *p* = 0.373, ηp^2^ = 0.07) (Figure 2A,B). Lean mass (MD: −0.64 ± 1.79 kg; *p* = 0.488, ηp^2^ = 0.041) nor VAT (MD: −0.31 ± 1.36 cm; *p* = 0.653, ηp^2^ = 0.011) resulted in no significant changes between groups (Figure 2C). 

For females, weight change was not significant for either the MRP group or CON group (MD: 0.67 ± 1.64 kg; *p* = 0.425). There was no significant difference in FM for the MRP or CON groups (MD: −0.73 ± 1.37 kg; *p* = 0.302, ηp^2^ = 0.66); there was also no difference in %BF between groups (MD: −0.57 ± 1.26%, ηp^2^ = 0.05). LM (MD: 0.31 ± 1.37 kg, *p* = 0.655, ηp^2^ = 0.013) and VAT (MD: −0.83 ± 1.2 cm, *p* = 0.167, ηp^2^ = 0.103) were also not different between groups. 

### 3.2. Metabolism

**Figure 3:**
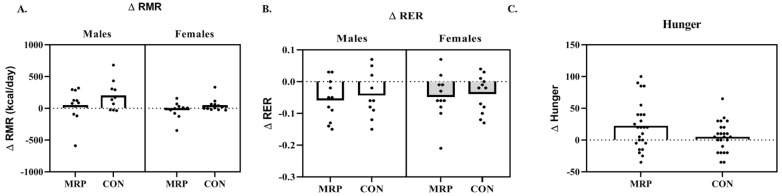
Individual responses for change in (**A**) resting metabolic rate (∆RMR); (**B**) resting respiratory exchange ratio (∆RER); and (**C**) hunger for the meal replacement (MRP) and control (CON) groups.

For males, there was no difference demonstrated for RMR (MD: −152.97 ± 219.40 kcals, *p* = 0.180, ηp^2^ = 0.097), RER (MD: −0.018 ± 0.06, *p* = 0.550, ηp^2^ = 0.020), or ∆RER (MD: −0.010 ± 0.04, *p* = 0.583, ηp^2^ = 0.017) between groups (Figure 3A,B). 

Females also demonstrated no difference for RMR (MD: −72.7 ± 92.18 kcals, *p* = 0.130, ηp^2^ = 0.111), RER (MD: 0.001 ± 0.04, *p* = 0.975, ηp^2^ = 0.001), or ∆RER (MD: −0.01 ± 0.03, *p* = 0.440, ηp^2^ = 0.033) between groups (Figure 3A,B). 

### 3.3. Metabolic Blood Markers

For men, there were no significant differences in any of the blood markers when compared to the control group (Table 2). However, MRP total cholesterol (MD: 20.02 ± 22.94 mg/dL; *p* = 0.098, ηp^2^ = 0.145) and LDL (MD: 23.98 ± 23.26 mg/dL; *p* = 0.054, ηp^2^ = 0.191) were increased from pre to post compared to the control group.

All blood markers in females remained consistent except for HDL. The MRP group demonstrated a significant reduction in HDL (MD: −6.41± 4.44 units, *p* = 0.018) compared to the control group. All other markers did not change. Although not significant, TRG values appeared to increase for the CON and remain stable for MRP (Table 2).

### 3.4. Nutrition

There were no significant baseline differences for total CAL, CHO, PRO, FAT, or CHO/(PRO + FAT) ratio for either men or women. Calories did not vary significantly between groups throughout the duration of the intervention; however in the MRP group, males and females consumed fewer calories (MD: −426 ± 541 kcals, *p* = 0.132; MD: −148 ± 373 kcals, *p* = 0.438, respectively) compared to the control group. There was no difference between groups for CHO (*p* = 0.186, *p* = 0.502), PRO (*p* = 0.095, *p* = 0.957) or FAT (*p* = 0.137, *p* = 0.297) for men and women, respectively. When evaluating the CHO/(PRO+FAT) ratio there was a significant interaction for males (*p* = 0.014) and females (*p* = 0.001). When decomposed, this ratio was significantly lower at week eight for the MRP groups in both men (*p* = 0.05) and women (*p* = 0.001), compared to the control group.

### 3.5. Questionnaires

There were no significant interactions or main effects (*p* > 0.05) for any of the items on the questionnaire regarding hunger. When males and females were combined, hunger during the afternoon was significantly reduced for MRP from “slightly hungry” (−22.86 ± 21.93) to “neither hungry nor full” (−3.86 ± 34.60; *p* = 0.021) pre-post, which was less than CON (Post: −22.38 ± 25.87; *p* = 0.054). Hunger during the afternoon did not change pre-post for CON (Pre: −26.90 ± 18.67; Post: −22.38 ± 25.87; *p* = 0.396) (Figure 3C). There were no changes right after breakfast or a few hours after breakfast (*p* > 0.05).

## 4. Discussion

Although previous studies suggest that high-fat, MCT consumption may lead to potential increases in energy expenditure and decreased body fat [10,11,27], results of the current study show that consumption of a high-fat, MCT breakfast meal replacement shake did not lead to significant changes in body composition, metabolism, or metabolic blood markers over the course of eight weeks. The effects were broadly similar between males and females; females saw a significant reduction in HDL (−6.44 units). High fat, MCT at breakfast, may have potential benefits for managing hunger and caloric intake. Individuals in the MRP group reported reduced hunger during the afternoon and tended to consume fewer calories on average, which may be beneficial for preventing weight gain, but more significant lifestyle changes, such as modifying exercise and caloric intake, as well as a longer duration may be required to promote weight loss and change in body composition.

Consumption of MCTs is thought to promote weight loss and improvements in body composition by increasing thermogenesis and promoting fat oxidation [5]. Various acute interventions ranging from a single meal to 24-h have reported greater fat oxidation, increased post-prandial thermogenesis, and increased energy expenditure following MCT consumption compared to long-chain triglycerides (LCT) [28,29,30,31,32,33]. Despite promising results from acute interventions, the long-term benefits are less consistent. When comparing the results of three crossover interventions, diets enriched with MCT increased energy expenditure and fat oxidation after a single use and after seven days, compared to a LCT enriched diet [10,11,31]. However, after 14 days, there were no longer significant differences between MCT and LCT diets. At 28 days, overweight men had greater decreases in body mass and total body fat when consuming a MCT rich diet, albeit a 1 kg and 0.8 kg loss respectively, despite non-significant differences in energy expenditure and fat oxidation [10]. In contrast, overweight women experienced no significant changes in body composition, but had slightly greater energy expenditure and fat oxidation with a MCT rich diet [11]. Other studies have reported greater changes in body composition [34], but results of the current eight-week intervention found no effects of MCT breakfast consumption on metabolic rate, fat oxidation, or body composition in overweight men or women. Lack of significant changes in the current study could be a result of the amount of MCT consumed. The MRP used in the current study was 155 kcal with 12.5g of fat consumed only at breakfast, compared to other interventions which dosed up to >20% total daily energy intake (54 g of MCT per day or ~18 g per meal) [5]. In a study by Dulloo et al. [30] greater MCT:LCT ratio (15–30g of MCT) was associated with greater energy expenditure, thus the daily MCT:LCT of the current study may not have been high enough to elicit metabolic and body composition adaptations [31]. Despite the lack of benefits of MCT consumption in the current study, results of a recent meta-analysis of interventions lasting ≥3 weeks show that MCT consumption may result in only an average body weight loss of 0.51 kg over the course of about 10-weeks, suggesting that the overall benefits of MCT consumption for the purposes of weight loss may not be robust and may require a longer amount of time to result in meaningful changes [5]. Additionally, there appeared to be no major effects on blood markers with the use of daily MRP ingestion. Female HDL values yielded a significant decrease from pre to post (−6.44 points), this decrease cannot be fully explained by the present intervention, but it may be attributed to small changes in physical activity that occurred throughout the eight weeks, although this was not measured. It may also be attributed to the larger coefficient of variation in the female sample compared to the males.

Despite no effects on body composition or metabolism, results of the current study do suggest a potential effect on hunger and satiety, specifically later in the day. It is unclear if this is due to breakfast consumption in general or greater MCT. Specifically, as total caloric intake was reduced in the MRP group (−426 ± 541 kcals) compared to CON (−148 ± 373 kcals). It may be insightful to compare the breakfast composition between treatment groups; the diet analyses were limited by the inability to truly differentiate what meal was considered breakfast. Fat is known for its positive effect on satiety [4]; but specific to breakfast, previous studies support the need for a dietary restraint in combination with a high fat breakfast for positive effects on satiety [4]. The present study also used a liquid meal replacement, which may lead to lower satiety than a solid meal replacement [34]; the minimal intervention provided in the present study still resulted in lower perceived hunger. Future studies looking specifically at physiological markers of hunger (i.e., ghrelin) are warranted to support or refute the present findings. Additionally, the potential influence of physical activity on these outcomes may be impactful; identifying exercise patterns and intensity in future studies is warranted.

## 5. Conclusions

This pragmatic trial aimed to make minor behavior changes to evaluate the effects of a minimal nutrition intervention at breakfast on body composition and metabolism. Broadly, there were no effects on body composition, metabolism, or lipids. These results may be limited by the lack of control of diet outside of breakfast consumption, as well as the lack of exercise tracking. All subjects were asked to maintain normal habits, which may be supported by the lack of change for either group. There was a positive effect on satiety in the afternoon, but this did not correspond to any direct benefit. Extending the duration of this minimal nutrition intervention beyond eight weeks or combining it with a lifestyle modification may prove to be more effective. A high-fat liquid breakfast does not appear to be advantageous for overweight and obese men and women. Additionally, a consistent use of a high-fat breakfast does not appear to negatively impact blood lipids. 

## Figures and Tables

**Table 1:** Demographic descriptive data for modified intention to treat analysis. Mean ± Standard Deviation.

Treatment	Age (yrs)	Height (cm)	BMI (kg∙m^−2^)	%fat
MRP (*n* = 22)	34.4 ± 9.5	170.7 ± 9.2	33.3 ± 4.9	37.2 ± 7.0
CON (*n* = 21)	36.2 ± 8.9	169.7 ± 10.7	33.8 ± 5.7	36.2 ± 8.9

**Table 2:** Raw data for the blood variables for the meal replacement (MRP) and control (CON) groups for total cholesterol (TC), high-density lipoproteins (HDL), low-density lipoproteins (LDL), triglycerides (TRG), and glucose (GLU) from before (pre) and after (post) eight weeks of supplementation. Data is presented as mean ± standard deviation.

	**TC (mg/dL)**		**HDL (mg/dL)**		**LDL (mg/dL)**		**TRG (mg/dL)**		**GLUC (mg/dL)**	
	**Pre**	**Post**	**Pre**	**Post**	**Pre**	**Post**	**Pre**	**Post**	**Pre**	**Post**
**Male**										
MRP	197.4 ± 40.1	213.9 ± 43.2	41.0 ± 7.7	40.9 ± 8.6	123.5 ± 32.5	141.0 ± 30.9	165.2 ± 117.9	160.6 ± 92.2	88.2 ± 11.1	90.1 ± 13.7
CON	182.2 ± 29.7	177.1 ± 50.2	36.8 ± 8.5	36.5 ± 10.4	121.9 ± 27.8	115.6 ± 42.8	117.4 ± 43.4	124.2 ± 54.7	99.5 ± 43.7	109.5 ± 3.5
**Female**										
MRP	167.6 ± 46.7	165.1 ± 42.9	49.0 ± 13.0	46.4 ± 11.6*	102.5 ± 38.9	103.5 ± 35.0	81.8 ± 46.5	76.5 ± 34.9	81.6 ± 5.6	85.5 ± 3.9
CON	180.7 ± 46.6	176.0 ± 45.9	47.4 ± 12.5	47.9 ± 16.0	116.7 ± 45.3	113.9 ± 37.0	82.0 ± 31.5	107.1 ± 51.6	82.0 ± 5.1	87.0 ± 9.6

* indicates a significant decrease compared to control from the ANCOVA analysis. *p* < 0.05.
